# Supplementary material for: Modulation of ER Stress and Inflammation by S-Ketamine, R-Ketamine, and Their Metabolites in Human Microglial Cells: Insights into Novel Targets for Depression Therapy
Source: Cells. 2025 Jun 3;14(11):831. doi: 10.3390/cells14110831 (PMC12154294; doi:10.3390/cells14110831)
Supplement: Supplementary file 1 [file cells-14-00831-s001.zip › cells-3633544-supplementary.pdf]

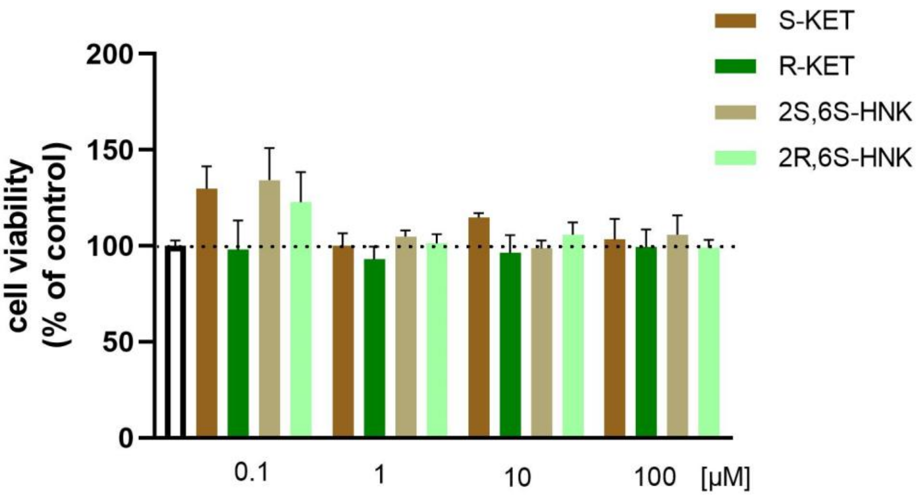

Supplementary Figure S1

Effects of S-ketamine (S-KET), R-ketamine (R-KET), and their metabolites (2S,6S-HNK and 2R,6R-HNK) on the viability of human microglial cells after 24-hour incubation. Cell viability was assessed using the MTT assay following treatment with increasing concentrations of each compound (0.1, 1, 10, and 100 µM). Data are presented as mean ± SEM and expressed as a percentage of untreated control cells (% of control). No significant cytotoxic effects were observed at any tested concentration.
